# Full-Length Recombinant *Plasmodium falciparum* VAR2CSA Binds Specifically to CSPG and Induces Potent Parasite Adhesion-Blocking Antibodies

**DOI:** 10.1016/j.jmb.2010.01.040

**Published:** 2010-04-02

**Authors:** Pongsak Khunrae, Madeleine Dahlbäck, Morten A. Nielsen, Gorm Andersen, Sisse B. Ditlev, Mafalda Resende, Vera V. Pinto, Thor G. Theander, Matthew K. Higgins, Ali Salanti

**Affiliations:** 1 Department of Biochemistry, University of Cambridge, 80 Tennis Court Road, Cambridge CB2 1GA, UK; 2 Centre for Medical Parasitology at Department of International Health, Immunology, and Microbiology, University of Copenhagen and at Department of Infectious Diseases, Copenhagen University Hospital (Rigshospitalet), Copenhagen, Denmark

**Keywords:** IE, infected erythrocyte, PfEMP1, *Plasmodium falciparum* erythrocyte membrane protein 1, CSA, chondroitin sulphate A, CSPG, chondroitin sulphate proteoglycan, GAG, glycosaminoglycan, DBL, Duffy-binding-like, PAM, pregnancy-associated malaria, CSPG-h, human chondroitin sulphate proteoglycan, FV2, full-length VAR2CSA, IMAC, immobilized metal ion affinity chromatography, IEX, ion exchange, AC, affinity chromatography, CSC, chondroitin sulphate C, HSPG, heparan sulphate proteoglycan, CSPG-b, bovine chondroitin sulphate proteoglycan, MFI, mean fluorescence intensity, malaria, PfEMP1, VAR2CSA, vaccine, CSA

## Abstract

*Plasmodium falciparum* malaria remains one of the world's leading causes of human suffering and poverty. Each year, the disease takes 1–3 million lives, mainly in sub-Saharan Africa. The adhesion of infected erythrocytes (IEs) to vascular endothelium or placenta is the key event in the pathogenesis of severe *P. falciparum* infection. In pregnant women, the parasites express a single and unique member of the *P. falciparum* erythrocyte membrane protein 1 (PfEMP1) family named VAR2CSA, which is associated with the ability of the IEs to adhere specifically to chondroitin sulphate A (CSA) in the placenta. Several Duffy-binding-like domains from VAR2CSA molecules have been shown *in vitro* to bind to CSA, but it has also been demonstrated that Duffy-binding-like domains from PfEMP1 proteins other than VAR2CSA can bind CSA. In addition, the specificity of the binding of VAR2CSA domains to glycosaminoglycans does not match that of VAR2CSA-expressing IEs. This has led to speculation that the domains of native VAR2CSA need to come together to form a specific binding site or that VAR2CSA might bind to CSA through a bridging molecule. Here, we describe the expression and purification of the complete extracellular region of VAR2CSA secreted at high yields from insect cells. Using surface plasmon resonance, we demonstrate that VAR2CSA alone binds with nanomolar affinity to human chondroitin sulphate proteoglycan and with significantly weaker affinity to other glycosaminoglycans, showing a specificity similar to that observed for IEs. Antibodies raised against full-length VAR2CSA completely inhibit recombinant VAR2CSA binding, as well as parasite binding to chondroitin sulphate proteoglycan. This is the first study to describe the successful production and functionality of a full-length PfEMP1. The specificity of the binding and anti-adhesion potency of induced IgG, together with high-yield production, encourages the use of full-length PfEMP1 in vaccine development strategies.

## Introduction

Infections are often initiated by the attachment of a pathogen to a host receptor present on the endothelium (reviewed by Yamada and Sugahara^1^). Understanding the structure–function relationship between pathogen ligand and host receptor is important for the design of effective vaccines and drugs targeting this interaction. *Plasmodium falciparum* malaria remains one of the leading causes of human suffering and poverty in the world (reviewed by Sachs and Malaney^2^). The adhesion of infected erythrocytes (IEs) to vascular endothelium or placental tissue is a key event in the pathogenesis of severe *P. falciparum* infection. By binding to receptors in the vascular bed, the parasites avoid being filtered through the spleen, where they are removed from the circulation.^3^ Adhesion of IEs to the vascular endothelium or the placental tissue is mediated by members of the *P. falciparum* erythrocyte membrane protein 1 (PfEMP1) family, which are encoded by the polymorphic multicopy *var* gene family.^4–7^ PfEMP1 proteins are large molecules (150–350 kDa) containing two to eight extracellular domains. During infection, parasites expressing PfEMP1 proteins mediate adhesion to a variety of human receptors. Among the best studied receptors are CD36, which is present in platelets and endothelial cells, and intercellular adhesion molecule-1, which is present in the endothelial lining (reviewed by Rowe *et al.*^8^). In pregnant women, the parasites express a single and unique variant of PfEMP1 named VAR2CSA, which enables IEs to adhere to the chondroitin sulphate A (CSA) chains of chondroitin sulphate proteoglycans (CSPGs) in the placenta but weakly to other glycosaminoglycans (GAGs).^9–11^

VAR2CSA is a large 350-kDa protein comprising six Duffy-binding-like (DBL) domains and three larger interdomain regions. Knockout studies of the *var2csa* gene have demonstrated the essential role of VAR2CSA in CSA binding, preventing the parasite from regaining the ability to bind to CSA.^12,13^ Antibodies to the surface-expressed VAR2CSA protein are acquired by women who are exposed to malaria during pregnancy, and high levels of anti-VAR2CSA antibodies at delivery are associated with protection from low-birth-weight babies,^10^ one of the major complications of pregnancy-associated malaria (PAM). Antibodies targeting VAR2CSA presumably abrogate or prevent binding to the vascular bed and thus protect against the adverse effects of the disease. VAR2CSA is recognized as the leading PAM vaccine candidate, and antibodies to single domains have, to varying degrees, been found to inhibit parasite binding to CSA.^14,15^

Several DBL domains from VAR2CSA molecules have been shown *in vitro* to bind to CSA.^16–20^ However, it has also been demonstrated that single DBL domains from other PfEMP1 proteins can bind CSA, although these proteins are not implicated in placental malaria.^20^ In addition, single DBL domains bound with roughly equal affinity to different glycans, whereas VAR2CSA-mediated binding of IEs is highly specific for CSA. Instead, it has been shown that the measured interaction between recombinant VAR2CSA domains and CSA *in vitro* is primarily electrostatic and independent of the structure of the GAG.^20,21^ This indicates that either the CSA-binding region of VAR2CSA comprises regions from multiple domains that come together only in the intact protein, with the possibility that some of the nonspecific binding regions exposed in recombinant domains are buried in native VAR2CSA, or the interaction between native VAR2CSA on the IE surface and CSA is mediated through another molecule.

Analysis of the structure–function relationship of PfEMP1 proteins has been hampered by technical difficulties in purifying native protein and the inability to express the entire extracellular region of PfEMP1 as recombinant constructs. Here, we describe the cloning and production of the first full-length extracellular part of a PfEMP1 protein, VAR2CSA, in a secreted and soluble form. This protein binds specifically and with high affinity to human chondroitin sulphate proteoglycan (CSPG-h) in a CSA-dependent manner and induces antibodies that are able to potently react with native VAR2CSA on IEs and inhibit the binding of IEs to CSPG.

## Results

### Cloning, production, and purification of the full-length extracellular part of VAR2CSA

**Fig. 1:**
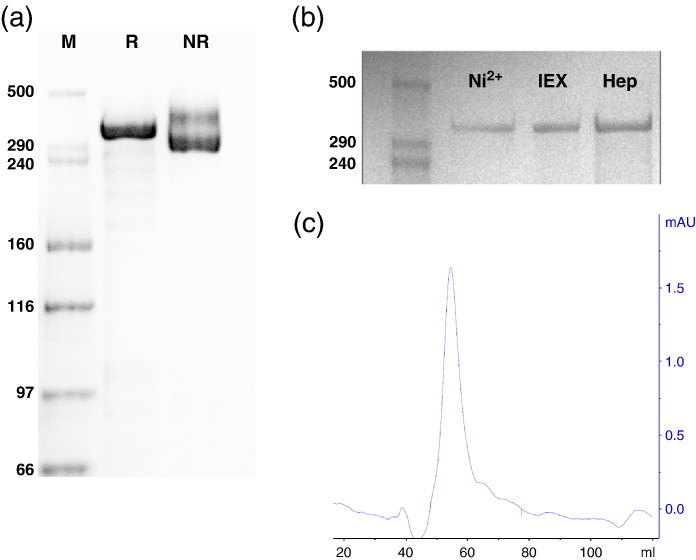
Production and purification of FV2. (a) SDS-PAGE of recombinant VAR2CSA (FV2) purified on an IMAC column: reduced (R) and nonreduced (NR). (b) SDS-PAGE of FV2 purified on a 1-ml HisSelect column (Ni^2+^), a 1-ml Capto S HP column (IEX), and a 1-ml Heparin HP column (Hep). Twenty microliters of a 1:240 dilution of each purification was loaded for comparison of yield. (c) Size-exclusion chromatography on a Superdex-200 column (GE Healthcare) of IMAC-purified FV2.

A synthetic *var2csa* gene was designed based on the new annotation of the *var2csa* gene from the FCR3 parasite genome‡. Full-length VAR2CSA (FV2) was cloned to allow translation from the N-terminal methionine (M1) to amino acid F2649, which is located before the putative transmembrane region. FV2 was secreted into the supernatant as a soluble protein (Fig. 1a), with only a small amount of protein detectable in cell pellets. FV2 protein was produced and secreted as a monomer (Fig. 1a and c). Nonreduced protein migrates on SDS-PAGE as two bands, which are reduced to a single band. The majority of the protein is present in the fastest migrating band, which is the size expected of a nonreduced protein. To examine whether the two folding variants observed in nonreduced SDS-PAGE were due to different glycosylation patterns, we produced the protein in the presence of tunicamycin, which blocks glycosylation. However, nonglycosylated FV2 also migrated as two bands, although of a smaller size (data not shown).

To compare the efficiency of three different protein-capturing techniques [immobilized metal ion affinity chromatography (IMAC), ion exchange (IEX), and affinity chromatography (AC)], we loaded equal amounts of culture supernatant on a 1-ml HisSelect column (IMAC), a 1-ml HiTrap Capto S column (IEX), and a 1-ml HiTrap Heparin HP column (AC), and we eluted bound protein with either imidazole (IMAC) or a NaCl chloride gradient (IEX and AC), as described in Materials and Methods. Figure 1b shows the purified protein, and we found that IMAC yields around 1 mg of FV2 per liter of culture supernatant, whereas IEX and AC yield around 2 mg/L and 4 mg/L, respectively. Gel filtration of the IMAC-purified protein shows that FV2 is monomeric also in the absence of SDS (Fig. 1c). The monomeric nature of FV2 was also confirmed by analytical ultracentrifugation studies (data not shown).

### GAG-binding properties of recombinant VAR2CSA

**Fig. 2:**
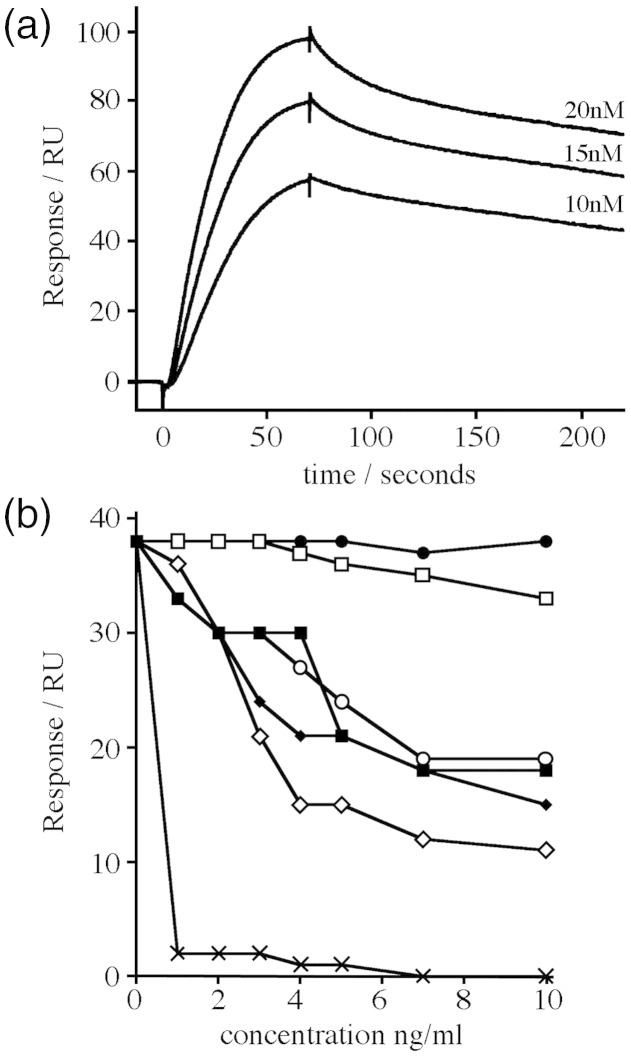
Recombinant VAR2CSA binds specifically to CSPG with subnanomolar affinity. (a) Human placental CSPG was coupled to the surface of a Biacore chip, and responses were measured after the injection of 10 nM, 15 nM, or 20 nM of the extracellular region of VAR2CSA (FV2) through the chip surface. (b) Competition experiments in which 10 nM FV2 was incubated with different concentrations of (●) hyaluronic acid, (□) HSPG, (♦) dermatan sulphate, (◊) CSA, (○) CSC, (▪) heparin, and (×) CSPG-b before the assessment of binding to a CSPG-h-coated Biacore chip surface.

In previous studies, we used a surface-plasmon-resonance-based assay to study the binding of individual domains from VAR2CSA to CSPG-h coupled to the surface of a Biacore chip.^21^ This revealed that the DBL3X and DBL6ɛ domains of VAR2CSA bind to CSPG-h with mid-micromolar affinities (33 μM for DBL3X and 80 μM for DBL6ɛ).^21^ Here, we passed different concentrations of recombinant FV2 over a similar CSPG-coupled surface, measuring the binding and dissociation of the protein from the chip. The data fitted to a one-to-one binding model with a *K*_d_ of 0.36 nM, an association constant of 7.3 × 10^6^ M^− ^^1^ s^− 1^, and a dissociation constant of 2.6 × 10^− 3^ s^− 1^ (Fig. 2a). Therefore, the intact extracellular part of VAR2CSA binds to CSPG-h with an affinity some 100,000-fold greater than that observed for single recombinant domains.

We next tested the GAG specificity of FV2. Previous studies have shown that the single DBL3X and DBL6ɛ domains from VAR2CSA lack the binding specificity observed for VAR2CSA-expressing IEs, with many GAGs able to compete for binding with CSPG. Indeed, the greater is the degree of sulphation, the more strongly a GAG competes.^21^ We performed a similar experiment to assess the binding specificity of VAR2CSA to CSPG-h. Seven different GAGs were used in these inhibition studies: hyaluronic acid from human umbilical cord, bovine CSA and chondroitin sulphate C (CSC), dermatan sulphate, heparan sulphate proteoglycan (HSPG), heparin, and decorin [bovine chondroitin sulphate proteoglycan (CSPG-b)]. These GAGs are different in terms of sulphation, negative charge, and glycan backbone structure. In each case, FV2 at a concentration of 10 nM was preincubated with different concentrations of GAGs before being passed over a CSPG-h-coated Biacore chip surface.

The various GAGs showed very different profiles in blocking the binding of FV2 to CSPG-h (Fig. 2b). Nonsulphated hyaluronic acid had no effect, and HSPG had very little effect, whereas the chondroitin sulphates (CSPG-b, CSA, CSC, and dermatan sulphate) and the most sulphated GAG (heparin) showed more significant inhibition. The strongest effects were detected with the CSA-containing GAGs bovine CSA and CSPG-b, with CSPG-b causing almost complete inhibition at 1 ng/ml. CSPG-b is a proteoglycan containing CSA chains that resembles human placental CSPG in structure and sulphation pattern.^22^ Therefore, the glycan that is most similar to the native CSPG showed the greatest affinity for recombinant FV2. This suggests that the ability to selectively bind to low-sulphated CSA chains observed for VAR2CSA-expressing IEs is due to the selectivity of the extracellular region of the VAR2CSA protein. While this does not rule out other CSA-specific proteins on the IE surface, these are not necessary to explain CSA-specific binding of VAR2CSA.

### Inhibition of the CSA binding of recombinant VAR2CSA using VAR2CSA-specific IgG

**Fig. 3:**
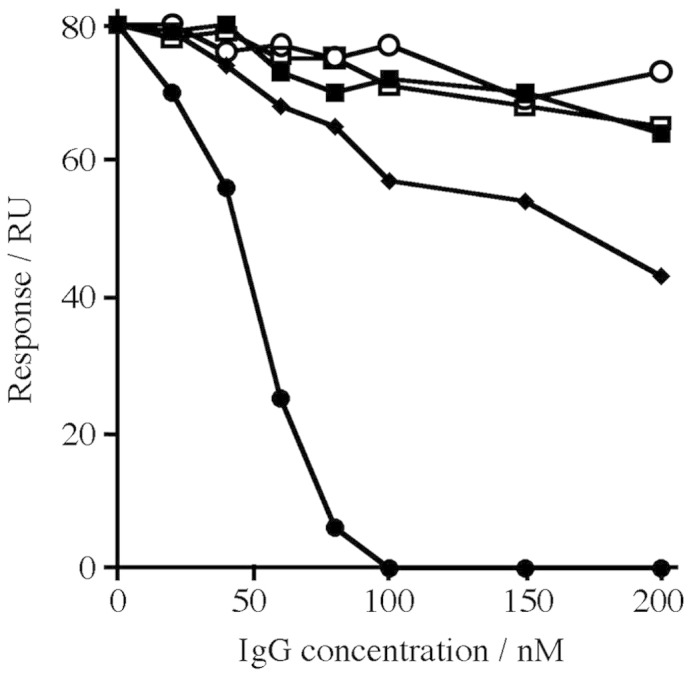
Purified IgG raised against recombinant VAR2CSA inhibits the binding of VAR2CSA to CSPG. The extracellular region of VAR2CSA (FV2) at a concentration of 10 nM was incubated with different concentrations of purified IgG raised against (●) FV2, (♦) DBL4ɛ domain of VAR2CSA, (▪) VAR1 DBL3γ, (□) prebleed, and (○) CIDR VAR4 before the assessment of binding to a CSPG-h-coated Biacore chip surface.

Antibodies were induced against the FV2 protein and against two control PfEMP1 domains. IgG was purified from immune and preimmune sera from the immunized animals. In addition, antibodies raised against the DBL4ɛ domain from VAR2CSA, which were previously found to be most effective in preventing the binding of IEs to CSPG-b,^14^ were purified and tested. These IgGs were tested for the ability to inhibit the binding of FV2 to CSPG-h using the surface plasmon resonance assay described above. In each case, FV2 at a concentration of 10 nM was preincubated with different concentrations of purified IgG before binding to a CSPG-h-coated surface was assessed. While the control IgG showed no effect on the ability of FV2 to bind to CSPG-h, IgG from rats immunized with the recombinant VAR2CSA protein inhibited this interaction, with complete inhibition at a total IgG concentration of 100 nM (Fig. 3). These IgGs were more effective than the antibodies raised against the single DBL4ɛ domain protein.

### Inhibition of IE binding to CSPG using VAR2CSA-specific antibodies

**Fig. 4:**
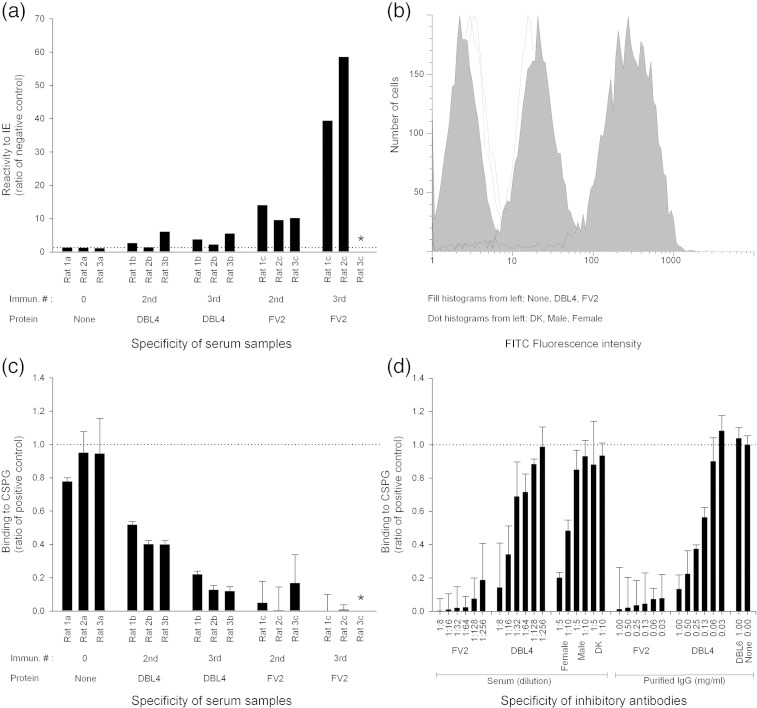
Reactivity against native VARCSA on the surface of FCR3 IEs. Groups of three rats were immunized with DBL4ɛ or FV2 recombinant proteins. The reactivity of the induced IgG was measured using flow cytometry and inhibition of binding assays. (a) Measurements of IgG levels in serum after two and three immunizations against native VAR2CSA expressed on IEs by flow cytometry. The reactivity shown is the ratio of negative controls (MFI test/MFI negative control). The negative controls were stained only by secondary fluorescein-isothiocyanate-conjugated anti-rat IgG (no rat IgG). The dotted line indicates the negative cutoff. (b) Examples of histograms of IEs (ethidium-bromide-positive cells) stained by IgG in sera. Filled histograms from the left: not immunized (rat 1a); DBL4ɛ (rat 3b after the third immunization), and FV2 (rat 2c after the third immunization). Dotted histograms from the left: Danish naïve pool, male immune pool, and female immune pool. (c) Inhibition of IE binding to decorin (CSPG-b) using serum in a 1:8 concentration. The dotted line indicates the level of no inhibition. (d) Left: Titration of inhibitory reactivity in serum pools of three rats or pools of serum from 10 individual naïve Danes, 10 Tanzanian males, and 10 Tanzanian females. Right: Titration of purified IgG from pools of three rats immunized with DBL4ɛ, FV2, or DBL6 (negative control). All error bars indicate standard errors of the means. *This rat was sacrificed for monoclonal IgG production before the third immunization.

To examine the reactivity of IgG induced against FV2 in rats, we used erythrocytes infected with the homologous FCR3 parasite line. Expression of VAR2CSA was continuously selected for by panning the IE on BeWo cells, as described previously.^23^ The resulting parasite line was recognized by malaria endemic plasma in a sex-specific manner and bound specifically to CSPG-b (data not shown). The reactivity of FV2-specific IgG was benchmarked against DBL4ɛ-specific serum and IgG. The DBL4ɛ domain was recently shown to be the construct that induces the strongest parasite-inhibitory response.^14^ In addition, we compared the reactivity to pools of human serum. We found a 14-fold higher recognition of the IEs by IgG in the FV2-specific serum compared to the DBL4ɛ-specific serum based on the mean fluorescence intensity (MFI) (Fig. 4a). The differences between the intensity of IgG staining using FV2-specific serum and that using monodomain-specific labeling and human pool labeling are exemplified in Fig. 4b. The functional capacity of the induced IgG to inhibit the binding of IEs to CSPG-b was measured in a static assay using a pipetting robot (Biomek; Beckman Coulter) to remove unbound IEs from CSPG-b-coated wells.

Binding in the presence of IgG from FV2-immunized rats was less than 10% of binding in media alone. Strongly inhibitory antibodies were already detectable after the second FV2 immunization. IgG from rats immunized with DBL4ɛ of VAR2CSA, the most potent inducer of inhibitory antibodies to date, showed considerably lesser activity than the anti-FV2 antibodies (Fig. 4c). This was further substantiated by comparing inhibitory activities in dilution series. Sera from FV2-immunized rats at a dilution of 1:256 inhibited to the same level as the serum from pregnant women at a dilution of 1:5, and anti-FV2 antibodies showed the same level of inhibition at 0.03 mg/ml as anti-DBL4ɛ antibodies at 1 mg/ml (Fig. 4d). Antibodies against FV2 were tested for the ability to inhibit a VAR2CSA-expressing heterologue parasite isolate (7G8) binding to CSPG-b. This was performed in a Petri dish assay using 1 mg/ml IgG, and bound IEs were stained and counted. Preliminary data showed that the FV2 antibodies inhibited 7G8 binding to CSPG-b by around 70% (data not shown).

## Discussion

The majority of pathogens that attach to the host endothelium seem to exploit the abundant and highly sulphated GAG heparan sulphate as a receptor (reviewed by Rostand and Esko^24^). By contrast, in malaria infection during pregnancy, the adhesion of IEs to intervillous spaces of the placenta is highly specific for CSA.^25^ Understanding the mechanism underlying this unique CSA interaction is central to the design of an optimal vaccine that can abrogate IE adhesion in the placenta and protect pregnant women against the adverse effects of PAM.

Until recently, it was believed that the domains of PfEMP1 proteins are ordered as ‘beads on a string,’ with different individual domains able to bind independently to receptor molecules. Based on this, the obvious approach for identifying the functional parts of PfEMP1 has been to analyze the binding properties of the corresponding domains expressed as single recombinant proteins.^14,15,26–28^ Consequently, this has been the main focus of PAM vaccine development, with considerable effort exerted to define a single domain of VAR2CSA that can be included in a vaccine. In the light of recently published data, it has become apparent that single domains of VAR2CSA do not show the CSA-specific binding associated with IEs isolated from placental tissue.^20,21^ The DBL2X, DBL3X, and DBL6ɛ domains that have been shown to bind CSA *in vitro* also bind very well to heparan sulphate and heparin, with DBL3X and DBL6ɛ binding with a higher affinity to heparin than to CSA. These findings have raised questions regarding the interaction between VAR2CSA and CSA, leading to the suggestion that the CSA-specific binding site of native VAR2CSA is due to higher-order arrangements of the domains or that other CSA-specific binding molecules are involved. Following this, the production of the whole extracellular part of VAR2CSA was a priority in attempts to unravel the true binding mechanism.

Here, we present for the first time the production of the entire extracellular part of VAR2CSA from the FCR3 parasite (FV2), expressed as a soluble secreted protein in a eukaryotic expression system. Sufficient yields were obtained to allow us to both study the GAG-binding properties of FV2 and test the ability of this protein to induce functional antibodies in rats. The FV2 protein bound to CSPG-h with an affinity of 0.36 nM, a factor 100,000-fold stronger than that observed for any single domain of VAR2CSA. In addition, this interaction was CSA-specific, with the low-sulphated CSA present in CSPG-b inhibiting the VAR2CSA–CSPG interaction to the greatest degree. The fact that FV2 can bind specifically to human placental CSPG with high affinity suggests that native VAR2CSA on the surface of IEs does indeed contain the determinants needed to specifically and directly interact with CSA and does not require a bridging molecule. In addition, these results imply that the CSA binding site is complex in the sense that it is composed of residues from several domains, which need to be assembled in a correctly folded VAR2CSA protein to form the CSA-specific binding site.

Rat antibodies induced by a recombinant DBL4ɛ protein from VAR2CSA of FCR3 are able to inhibit IE binding to the CSA of the homologous parasite FCR3 by 90%.^14^ Since the CSA-binding region of VAR2CSA has not been defined, it is not clear how the inhibitory DBL4ɛ antibodies act to abrogate the interaction with CSA. These antibodies may work by targeting a few critical residues in the DBL4ɛ domain that is part of the CSA binding site, by blocking access to a neighboring binding site, or by preventing the formation of a higher-order structure of VAR2CSA needed to form the CSA binding pocket. In this case, introduction of a DBL4ɛ-based vaccine might lead to a strong selection for CSA-binding parasites with different amino acids in these positions in DBL4ɛ. Accordingly, it would be preferable to target highly conserved residues—or as many residues as possible—that are involved in receptor–ligand interaction to reduce the possibility of creating a population of parasites that have mutated in the receptor binding site without losing the ability to bind to the receptor. The FV2 construct, which contains the whole CSA binding site, will be particularly useful in overcoming these challenges. As presented in this study, the FV2 protein induces a highly potent and robust humoral response in rats, and FCR3 IE binding to CSA is completely blocked by these antibodies, which also seem to be cross-reactive with a heterologue isolate. These results are promising for vaccine development and open the way to future studies that will include analysis of the breadth of the antibody response, the mechanism of inhibition, and the development of protocols for the optimization of protein production to increase the yield of this large antigen.

## Materials and Methods

### *P. falciparum* cultures

Parasite culture was grown as previously described.^29^ In brief, parasites were maintained in a culture using 5% hematocrit of human blood group O^+^ blood in a parasite medium consisting of RPMI 1640 supplemented with 25 mmol/L sodium bicarbonate (Sigma-Aldrich), 0.125 μg/ml gentamicin, 0.125 μg/ml Albumax II (Invitrogen), and 2% normal human serum. To select for VAR2CSA expression, we repeatedly panned IEs on BeWo cells, as described previously.^23^ All isolates were negative for mycoplasma and were regularly genotyped using nested GLURP and MSP-2 primers in a single PCR step.

### Codon optimization and cloning

The FCR3 *var2csa* gene was optimized for expression in *Trichoplusia ni* cells using the software GeneOptimizer§ and subsequently synthesized by Geneart (Regensburg, Germany). Briefly, subgenetic fragments were assembled from synthetic oligonucleotides using a single-step PCR-based method. The full-length synthetic and optimized DNA was fused by ligation of the respective subgenetic fragments and cloned, and the insert was verified by sequencing. The codon-optimized gene sequence is deposited at GenBank with accession no. GU249598. The *var2csa* gene was subcloned into the baculovirus vector pAcGP67-A (BD Biosciences) modified to contain a V5 epitope upstream of a His tag at the C-terminal end of the construct. Linearized Bakpak6 Baculovirus DNA (BD Biosciences) was cotransfected with pAcGP67-A into Sf9 insect cells for the generation of recombinant virus particles. The control PfEMP1 proteins used to induce control IgG for the binding assay were based on 3D7 VAR4 CIDR (PFD1235w) and FCR3 DBL3γ, as defined by Nielsen *et al.*^14^

### Protein expression and purification

High-Five insect cells grown in 600 ml of serum-free media (10486; GIBCO) were infected with 18 ml of the second amplification of the recombinant virus particles. After 2 days of induction, the cells were centrifuged (8000***g***, 4 °C, 10 min), and the supernatant was filtered using two 10-kDa NMWC PES membranes (0.45 μm) (56-4112-04; GE Healthcare) with a total surface area of 200 cm^2^. The supernatant was then concentrated to 30 ml and diafiltrated six times on an ÄKTA cross-flow (GE Healthcare) with buffer A [10 mM sodium phosphate (pH 7.4) and 500 mM NaCl]. Retentate was recovered from the system and filtered (0.2 μm), yielding a final volume of 40 ml. A total of 10 ml of the retentate was loaded onto a 1-ml HisSelect column (H8286; Sigma-Aldrich), a 1-ml Capto S HP column (17-5441-22; GE Healthcare), and a 1-ml Heparin HP column (17-0406-01; GE Healthcare). Before loading on the HisSelect column, 150 μl of 1 M imidazole (pH 7.4; Sigma-Aldrich) was added to the sample, giving a final imidazole concentration of 15 μM. The samples for the Capto S HP and Heparin HP columns were diluted 1:10 with 10 mM sodium phosphate (pH 7.4), giving a final salt concentration of 50 mM NaCl. The bound protein was eluted with buffer A + 200 mM imidazole (HisSelect) or a salt gradient ranging from 50 mM to 1000 mM NaCl (Capto S HP and Heparin HP).

### Rat immunizations and IgG preparations

Rat anti-sera were produced by injection of 30 μg of recombinant protein in Freund's complete adjuvant, followed by two booster injections of 15 μg of protein in Freund's incomplete adjuvant at 3-week intervals. Anti-sera were collected 8 days after the final boosting injection. All procedures regarding animal immunizations complied with European and national regulations. All immunizations induced antibodies against the recombinant proteins, as measured by ELISA of the final bleed. IgG was purified by manually passing 0.5 ml of rat immune serum through a column packed with Recombinant GammaBind™ G type 2 coupled to Sepharose™ 4B, in accordance with the manufacturer's recommendations (GE Healthcare), and bound IgG was eluted with Tris–glycine (pH 2.4) and dialyzed against phosphate-buffered saline. Anti-DBL4ɛ IgG was the same IgG used by Nielsen *et al.*^14^

### Flow cytometry

Flow cytometry was used to test the reactivity of rat serum to VAR2CSA on the surface of the IEs. In brief, parasite cultures were enriched to contain late-trophozoite-stage and schizont-stage parasites by exposure to a strong magnetic field. Aliquots (2 × 10^5^ IE) were labeled with ethidium bromide and sequentially exposed to 10 μl of rat serum and 1 μl of anti-rat IgG FITC (Zymax; Invitrogen). Data were acquired using an FC500 flow cytometer (Beckman Coulter). All samples relating to a particular parasite isolate were processed and analyzed in a single assay.

### Parasite binding assays

Parasite binding assays with FCR3 IEs were performed as previously described.^29^ Briefly, 2 × 10^5^ tritium-labeled late-stage IEs and 15 μl of rat serum or IgG in a total volume of 100 μl were added in triplicate to wells coated with 2 μg/ml of the commercially available CSPG, decorin (CSPG-b; D8428; Sigma-Aldrich). The binding to CSPG-b was abrogated by soluble CSA (C9819; Sigma-Aldrich) and chondroitinase treatment (data not shown). After incubation for 30 min at 37 °C, unbound IEs were washed away by resuspension performed by a pipetting robot (Beckman Coulter). The proportion of adhering IEs was determined by liquid scintillation counting on Topcount NXT (Perkin-Elmer).

### Surface plasmon resonance measurements

Measurements were performed on a Biacore T100 instrument with a constant flow rate of 30 μl/min. Biotinylated CSPG-h was obtained from MR4 (deposited by C. Gowda) and coupled to an streptavidin chip (Biacore). CSPG-h was dissolved to a concentration of 10 μg/ml in HBS buffer [10 mM Hepes (pH 7.4), 150 mM NaCl, 50 μM ethylenediaminetetraacetic acid, and 0.05% Tween 20] and flowed through channel 2 until the response had increased by 400 RU. Flow cell 1 was left as a control surface.

Proteins were equilibrated into HBS buffer using PD10 columns (Amersham Biosciences). Both channels were equilibrated with HBS buffer before the injection of purified VAR2CSA protein. The level of specific binding was obtained from the subtraction of the response from channel 2 from that from channel 1. After each injection, both channels were regenerated with a 30-μl injection of 1 M NaCl. This procedure led to the recovery of the original baseline and regenerated a CSPG-h surface that could bind reproducibly to subsequent injections of FV2. Data were analyzed using the BIAevaluation software (version 1.1.1) to fit the kinetics of association and dissociation.

For competition experiments, GAGs were obtained from Sigma-Aldrich: CSA from bovine trachea (C9819), CSC from shark cartilage (C4384), dermatan sulphate from porcine intestinal mucosa (C3788), heparin from porcine intestinal mucosa (H9399), hyaluronic acid from human umbilical cord (53750), and HSPG from mouse sarcoma (H4777). Human placental CSPG is difficult to obtain in sufficient amounts for inhibition assays and parasite binding assays; therefore, the commercially available CSPG, decorin (CSPG-b), from bovine articular cartilage (D8428) was used in some experiments. Each GAG was dissolved in HBS buffer up to 1 mg/ml, and these stocks were mixed with the FV2 protein to produce samples with a final concentration of 1 nM FV2 and a range of GAG concentrations (1–10 ng/ml) in HBS buffer. These were incubated for a minimum of 30 min, and Biacore measurements were taken as described above.

## Footnotes

‡ Available at Sanger.ac.uk

§ http://www.geneart.com
